# Quality of education, ageing and labor productivity

**DOI:** 10.1371/journal.pone.0314367

**Published:** 2024-12-11

**Authors:** Fange Meng, Xin Wen

**Affiliations:** 1 School of Economics, Capital University of Economics and Business, Beijing, China; 2 National Academy of Innovation Strategy, China Association for Science and Technology, Beijing, China; Universita degli Studi del Piemonte Orientale Amedeo Avogadro, ITALY

## Abstract

With the decrease in fertility rate and the extension of life expectancy, China’s ageing degree is deepening, and there is a decrease in the number of labor force individuals, leading to an increase in the burden of old-age care and constraining economic growth. The improvement of human capital can promote economic growth. Research is rquired to determine whether factors such as the teacher-student ratio (quality of education) and the average number of years of schooling (quantity of education) can help alleviate the negative impacts of ageing. The findings demonstrate that education, both in terms of quantity and quality, can successfully reduce the detrimental consequences of ageing. The threshold effect model’s findings indicate that both the amount and quality of education can be more effective in reducing the negative impacts of ageing when average years of education surpass 10.87 years and the teacher-student ratio hits 7.80 (780 instructors per 1000 pupils). The results of heterogeneity analysis reveal that both the quantity and quality of education could potentially mitigate the negative effects of ageing in the eastern and western regions, although these factors do not seem to have the same effect in the central region. In the northern and southern regions, it is found that while the quantity of education can help alleviate the negative effects of ageing, the quality of education is effective only in the southern region and not in the northern region. Therefore, one potential strategy to counteract the adverse effects of ageing with a declining number of children is to increase the teacher-student ratio and extend the duration of free education.

## 1. Introduction

Labor productivity and labor force are important factors affecting economic growth. China is in the new normal period, with a slowdown in economic growth and changes in the age structure of the population. The United Nations refers to countries and regions where the proportion of elderly people aged 65 and above exceeds 7%, 14% and 20% respectively as ageing society, deeply ageing society and super-ageing society. According to calculations based on data from the National Bureau of Statistics, China’s total population at the end of the year was 1.267 billion in 2000, with the proportion of elderly people aged 65 and over at 6.96% and the proportion of people aged 0–14 at 22.89%. In 2001, the proportion of elderly people aged 65 and over had reached 7.10%, and the proportion of people aged 0–14 had reached 22.50%, which means that, according to international standards, China has begun to enter into an ageing society. In 2021, the proportion of elderly people aged 65 and above reach 14.2%, and China has formally entered a deeply ageing society. With the deepening of ageing, the number of labor force is declining in the process of demographic transformation, so improving labor productivity is particularly important for economic growth. At the Fourth Session of the 13th National People’s Congress, the target for the 14th Five-Year Plan period was to make the growth of total labor productivity higher than the growth of GDP. As life expectancy increases and the total fertility rate declines, it will be difficult to reverse the trends of ageing. China is faced with the shortage of labor supply, the increase of dependency burden, the slowdown of human capital improvement, the decline of savings rate and other unfavorable factors brought by ageing. The global labor supply is declining, and ageing may have a more serious impact on China ’s economic growth if measures are not taken in time. Therefore, it is of great significance for China to study how to cope with the adverse effects of ageing on labor productivity.

Human capital is an important factor in improving labor productivity. With the gradual disappearance of China’s demographic dividend and the arrival of Lewis turning point(Lewis turning point is the transfer of surplus labor force from agricultural sector to non-agricultural sector and the turning point from surplus labor force to shortage of agricultural sector.), the quality of labor force is receiving more and more attention, and the importance of human capital is increasing. Human capital formation mainly comes from education. Most literature studies on the relationship between human capital, ageing and labor productivity only consider the average years of education. Behrman and Birdsall [1] proposed the concept of effective education. Labor productivity is influenced by the length of years of education on average, which is determined by the quality of education. The higher the quality of education, the greater the effect of increasing average years of education on labor productivity [2]. In recent years, some studies have found a significant decline in the relative importance of education in explaining inter-regional income differences [3, 4]. Disregarding the quality of education can lead to an underestimation of the level of human capital [5, 6].

Encouraging economic growth is the most essential way to mitigate ageing. The quantity and quality of education can significantly increase labor productivity. We focus on whether the quality and quantity of education can mitigate the negative impact of ageing. With limited educational resources, it provides empirical evidence for whether different regions should choose to prioritize the development of education quality or the expansion of education quantity. The contribution of this paper lies in: we consider the quality of education when studying the role of human capital in mitigating the negative effects of ageing. Existing literature studies on measures to cope with ageing only consider the improvement of average years of education, ignoring the impact of education quality on labor productivity. The innovation of this research perspective provides a basis for the government to formulate education policies. Quantitative analysis of the impact of the number of education and the quality of education on the relationship between ageing and labor productivity, and estimated the threshold value of the number of education and the quality of education to maximize the role. It provides a basis for the government to formulate policies to extend the length of free education and increase the teacher-student ratio.

## 2. Literature review

The impact of ageing on economic growth in the long term cannot be ignored. China’s total fertility rate has fallen below the warning line, the growth of the child population has slowed down, and the growth rate of the elderly population is fast. The problem posed by ageing is not only "ageing before getting rich" but also, to a large extent, "ageing before getting ready". The impact of population ageing on labor productivity is different due to different labor substitution elasticity [7], and different industrial regions. Optimists argue that cross-country data prove that the rate of ageing has to reach a certain range before it has an impact on economic growth [8]. As the labor force ages, people are compelled to focus on developing labor-saving technology, human capital, and advancing technical advancement [9, 10]. The rich experience of older people is conducive to improving labor productivity [11, 12]. Some scholars believe that there is a non-linear relationship between ageing and labor productivity [13]. There is an inverted "U" structure between the labor force of each age group and its contribution to labor productivity [14, 15]. Li [16] argues that in the short run, ageing suppresses labor productivity in the current period and promotes labor productivity in the next period, but has no significant effect in the long run. Pessimists believe that the deepening of ageing is an important reason for the decline in productivity [17, 18]. China’s demographic dividend gradually weakens as a result of ageing-related drops in employment, increases in labor costs, and rising salaries. The number of surplus labor transferred from the agricultural sector has decreased, the Lewis turning point has emerged, and the efficiency of labor reallocation has decreased [19], which is not conducive to the improvement of labor productivity. With longer life expectancy and lower fertility rates, the negative impact of ageing on labor productivity is becoming more and more obvious. The key to achieving high-quality economic development lies in improving labor productivity [20].

To study how to improve labor productivity under ageing conditions, the first problem to be solved is how to alleviate the negative impact of ageing. There are literature studies on the potential of labor supply from the aspects of delaying retirement [21], adjusting fertility policy [22], and promoting household registration reform. There is also a part of the literature that studies methods such as artificial intelligence to change labor demand [23]. However, this literature rarely emphasizes the role of human capital in mitigating the ageing problem.

Much of the established literature focuses on education when examining the relationship between human capital, ageing and labor productivity. The level of education can determine the degree of technological innovation and technology diffusion [24]. It is easier for higher education to accept new knowledge and technology and improve productivity. High-quality human capital has higher production allocation capacity, which drives technological innovation [25] and higher labor productivity. Higher human capital can increase the effective labor supply and compensate for the negative impact of the decline in the number of labor force. Measuring human capital in terms of average years of education, the higher the level of education, the more valuable the work experience is. Education can increase labor productivity through experience [26], and the expansion of the quantity of education is conducive to labor productivity growth [27]. Workers with high levels of education have stronger personal learning ability and adaptability, and labor productivity is higher [28, 29]. Longer average years of education and higher knowledge absorption capacity are conducive to the improvement of labor force quality and the shift from relying on the population quantity dividend to relying on the population quality dividend. A second demographic dividend from human capital can offset the ageing-related reduction in the labor supply.

There is also literature on the impact of education quality on economic growth. Scholars mostly use the pupil/teacher ratio [30], the returns to schooling of foreign-educated immigrants in the U.S. [6], and international test scores measure the quality of education [31, 32]. The quality of education affects income. Card and Krueger [30] find that men have higher returns to additional years of schooling in areas with high quality of education, and that individual returns are higher in areas with higher numbers of highly educated teachers. Kaarsen [32] argues that the quality of education varies from country to country, which leads to differences in income among countries. High-quality education is an expression of technological progress [33]. Differences in the quality of education are also an important cause of differences in output per unit of labor [6]. The quality of education can contribute to economic growth, and even the quality of education contributes more than the quantity of education [31].

This paper finds that most of the literature studies the relationship between ageing, human capital and labor productivity from the perspective of the quantity of education, ignoring the impact of the quality of education. Under the condition of ageing, it is important to study whether the quantity and quality of education can alleviate the negative impact of ageing on labor productivity, which can help to narrow the gap in economic development between regions and achieve common prosperity.

## 3. Research design

### 3.1 Empirical model construction

The cross-multiplier terms of education quantity and education quality with ageing are added to the econometric model to establish the moderating effect model. This allows us to test whether education can counteract the negative effects of population ageing on labor productivity.


$$pgdp_{it}=\alpha_{0}+\alpha_{1}old_{it}+\alpha_{2}H_{it}+\alpha_{3}old_{it}\times H_{it}+\beta X_{it}+\mu_{i}+\eta_{t}+\varepsilon_{it}$$ (1)

In Eq (1), subscripts i, t denote regions and years, respectively. *pgdp*_*it*_ denotes labor productivity, *old*_*it*_ denotes the ageing ratio, *H*_*it*_ denotes human capital measured by the quantity and quality of education, *old*_*it*_×*H*_*it*_ is the cross-multiplier of ageing and human capital, *X*_*it*_ is a set of control variables including the share of children in the population, healthy human capital, physical capital per capita, technological level, industrial structure, and urbanization rate. *μ*_*i*_ and *η*_*t*_ denote area fixed effects and year fixed effects, respectively, and *ε*_*it*_ is a random error term.

The new geographical economics suggests that economic development is influenced not only by economic factors in the region, but also by economic factors in neighboring regions. Neglecting the correlation between regions may make the econometric model endogenous. A spatial Durbin model is developed to further validate the role of education quantity and quality in mitigating the negative effects of ageing under the condition of considering spatial factors:

$$\begin{array}{l} pgdp_{it}=\alpha_{0}+\rho_{0}Wpgdp_{it}+\alpha_{1}old_{it}+\alpha_{2}H_{it}+\alpha_{3}old_{it}\times H_{it}+\beta X_{it}\\ \;+\rho_{1}Wold_{it}+\rho_{2}WH_{it}+\rho_{3}Wold_{it}\times H_{it}+\beta WX_{it}+\mu_{i}+\eta_{t}+\varepsilon_{it} \end{array}$$ (2)

W in Eq (3) represents the spatial weight matrix. In this paper, we use three spatial weight matrices, which are inverse distance spatial weight matrix W_1_, economic geographic spatial weight matrix W_2_, and economic spatial weight matrix W_3_. In the inverse distance spatial weight matrix W_1_, the diagonal elements are all 0, and the element wij is the reciprocal of the distance between regions. The economic-geographic spatial weight matrix W_2_ is the product of the inverse distance spatial weight matrix W_1_ and the diagonal matrix where the mean value of the share of real GDP of each region is the diagonal element, that is, $W_{2}=W_{1}\times diag(\frac{{\bar{y}}_{1}}{\bar{y}},\ldots ,\frac{{\bar{y}}_{n}}{\bar{y}})$, where ${\bar{y}}_{i}=\frac{1}{t_{1}-t_{0}+1}\sum_{t=t_{0}}^{t_{1}}y_{it}$, $\bar{y}=\frac{1}{n(t_{1}-t_{0}+1)}\sum_{t=t_{0}}^{t_{1}}\sum_{i=1}^{n}y_{it}$. The economic spatial weight matrix W_3_ has the elements when *i*≠*j*, *pY*_*i*_ is the real GDP per capita of each region; and *w*_*ij*_ = 0 when *i* = *j*. When Eq (1) verifies the effect of education quantity and education quality on mitigating the negative impact of ageing, if *α*_3_ is significant, it means that education quantity and education quality can have an impact on the relationship between ageing and labor productivity. Then the threshold panel model is further developed to analyze the impact of ageing on labor productivity under different levels of human capital:

$$pgdp_{it}=\alpha_{0}+\alpha_{1}old_{it}\cdot I(H\leq \gamma )+\alpha_{2}old_{it}\cdot I(H>\gamma )+\beta X_{it}+\varepsilon_{it}$$ (3)

Eq (3) is an indicator function that takes the value of 1 when the condition in the parentheses holds, and 0 otherwise.

### 3.2 Data sources and descriptions of variables

The dependent variable is labor productivity. The value that a unit of labor contributes to production activities is known as labor productivity, or the effectiveness of a unit of labor involved in production. The gross domestic product divided by the number of employed people is the formula used to express labor productivity. Gross domestic product is converted to real values with 2000 as the base period.

The core explanatory variable is ageing. Ageing is defined as the proportion of people aged 65 and up in the overall population.

The quantity and quality of schooling serve as moderating variables for human capital. The quantity of education is measured by the average number of years of schooling. There are five levels of education, illiterate, elementary school, middle school, high school, college and above. The sum of each level of education and the accompanying length of schooling is expressed as a percentage of the employed population. The average years of schooling = (illiterate *0+ elementary school enrollment *6+ middle school enrollment *9+ high school enrollment *12+ college and above enrollment *16)/employed population. The middle school teacher-student ratio(%) is selected to represent the quality of education received. The teacher-student ratio is a measure of the quality of education. In general, the higher the teacher-student ratio, the greater investment in education, the better the corresponding infrastructure needs to be equipped, and the higher the quality of education [2]. The reasons for choosing the teacher-student ratio in middle school as an indicator of education quality are as follows: First, middle school is a critical period for students’ physical and mental development, and there is a strong correlation between the teacher-student ratio in middle school and students’ academic achievement, social skills and mental health. Second, middle school is directly related to higher education, and more emphasis is placed on the quality of education at the middle school stage in the education policy and evaluation system. Therefore, this paper chooses the middle school stage as the index to measure the quality of education.

Other control variables. Lower fertility affects the amount of future labor supply. The health of the labor force is directly related to productive life and affects labor productivity. Rural-urban migration brings labor to cities and also improves labor allocation efficiency, which can increase labor productivity. Technological progress is an important factor in promoting labor productivity. The proportion of employed persons in the total population affects labor productivity. On the one hand, the number of years of education for employed people who have participated in the work has been determined. The proportion of employed persons in the total population is taken as the control variable, and the influence of education quality on labor productivity is studied when the quantity of education is unchanged. On the other hand, the education stage of the employed personnel has been completed, and the quality of education has been established. The proportion of employed persons in the total population is taken as the control variable, and the influence of the number of education on labor productivity is studied when the quality of education is unchanged. Therefore, low fertility, health human capital, per capita physical capital stock, urbanization rate, industrial structure, the proportion of employed persons in the total population and technology level are chosen as control variables. Less-childrenization is measured by the ratio of the number of people aged 0–14 to the total number of people. Health human capital, expressed as the number of beds in medical institutions per 1,000 people. Per capita physical capital stock is calculated using the perpetual inventory method of Zhang et al. [34], which uses the formula in which the investment in the current year is discounted to the year 2000 as the base period, and the depreciation rate is set at 9.6%. The ratio of the permanent population in the region to the urban population is known as the urbanization rate. The percentage of secondary industry’s output value is used to measure industrial structure. The intensity of R&D spending is used to describe technology level.

Panel data from 31 areas between 2000 and 2020 are used in the analysis; the regional data come from the China Labor Statistics Yearbook, China Population and Employment Statistical Yearbook, and China Statistical Yearbook. The descriptive statistics of the variables are shown in Table 1. The mean and median of all variables are close to each other, indicating that the data of each variable are close to normal distribution or nearly normal distribution, which can better satisfy the conditions for the application of the econometric model. The difference between the maximum and minimum values of labor productivity is about 36 times, with the minimum value appearing in Guizhou in 2001 and the maximum value in Shanghai in 2020, and there is a large gap in labor productivity between regions. Most of the regions with a high proportion of elderly population and a low proportion of child population are in the Northeast and Eastern regions. There is a big difference between regions in the average years of education of the employed population. The average education level of the employed population in Beijing and Shanghai in 2020 reaches 13 years, which is equivalent to more than high school and college education, while the average years of education of the employed population in Xizang in 2020 is less than 7 years, which is equivalent to only junior high school education. The difference between the maximum and minimum teacher-to-student ratios for junior middle schools is about three times, and there are obvious differences in the quality of education between regions. From a regional perspective, labor productivity, ageing degree, average years of schooling and teacher-student ratio in the eastern region are higher than those in the western and central regions, and there may be regional heterogeneity in the quality and quantity of education to cope with the negative effects of ageing.

**Table 1:** Descriptive statistics of variables.

Variant	Symbol	N	Mean	St.D	Mini	Median	Max
**labor productivity**	pgdp	651	4.34	3.19	0.54	3.51	19.75
**ageing**	old	651	9.40	2.43	4.48	8.99	17.42
**quantity of education**	edu	651	9.14	1.53	3.02	9.11	14.52
**quality of education**	eduq	651	6.33	1.50	3.88	6.12	32.33
**lower fertility**	young	651	18.11	4.70	7.56	18.30	31.19
**urbanization rate**	city	651	51.27	15.92	18.93	50.50	89.58
**per capita physical capital stock**	pc	651	13.60	11.33	1.14	10.51	86.84
**industrial structure**	secp	651	42.15	8.35	15.97	43.02	61.96
**health human capital**	hd	651	4.09	1.57	1.48	3.97	7.95
**technology level**	RD	651	1.36	1.14	0.12	1.04	7.41
**the proportion of employed persons in the total population**	eml	651	55.18	6.29	36.37	55.31	76.03
**Regional heterogeneity:**							
**eastern region**	pgdp	231	6.51	3.86	1.37	5.66	19.75
	old	231	10.29	2.31	6.17	10.00	17.42
	edu	231	9.09	1.07	7.34	8.86	12.21
	eduq	231	6.43	1.16	4.31	6.30	10.21
**central region**	pgdp	168	3.25	1.75	0.91	2.92	8.94
	old	168	9.46	2.09	5.56	8.99	15.61
	edu	168	8.57	0.59	6.98	8.60	20.03
	eduq	168	6.13	0.88	4.14	6.11	7.93
**western region**	pgdp	252	3.07	1.99	0.54	2.63	10.60
	old	252	8.55	2.46	4.47	8.17	17.08
	edu	252	7.69	1.19	3.43	7.89	9.84
	eduq	252	6.38	2.01	388	6.03	32.33

## 4. Estimated results

A data stationarity test was conducted before performing the basic regression. According to the LLC test, IPS test, the results showed that the data were smooth.

### 4.1 Moderating effects of quantity and quality of education

The influence of ageing on labor productivity is tested using an econometric model based on Eq (1) to see if human capital, as indicated by the amount and quality of schooling, modifies the effect. Table 2 presents the results of the regression of demographic transition on labor productivity. The findings of the mitigating effect of education quantity on the negative impact of ageing are shown in column (1) in Table 2. The cross-multiplier term between ageing and average years of schooling is significantly positive, indicating that education quantity can reduce the negative impact of ageing on labor productivity. Ageing reduces the quantity of labor supply, and the arrival of the Lewis inflection point decreases the speed and quantity of labor transfer from rural to urban areas, reduces allocation efficiency, and is not conducive to the improvement of labor productivity. Older people and children only consume and do not engage in productive activities. Lower fertility rates, further ageing, and increased dependency burdens are detrimental to capital accumulation and capital deepening, thus lowering labor productivity. The cross-multiplier term between average years of schooling and ageing is positive, indicating that longer average years of schooling can mitigate the negative effects of ageing. An increase in average years of schooling is conducive to improving the quality of the employed population, increasing the effective supply of labor, and making up for the insufficient quantity of labor supply. The higher the education level, the easier it is to accept new technologies, and the increase in average years of education is conducive to the mobility of labor between regions and industries, improving allocation efficiency and increasing effective labor supply, thus alleviating the negative impact of ageing on labor productivity. High average years of schooling and high rate of return on human capital are conducive to capital accumulation and higher labor productivity.

**Table 2:** Moderating effects of human capital on ageing.

	(1)	(2)	(3)	(4)
	edu	eduq	edu	eduq
**old**	-1.4759***	-0.1615**	-0.9326***	-0.1170***
	(-11.0189)	(-2.4215)	(-9.8444)	(-2.4488)
**H**	-0.8693***	-0.0772	-0.6891***	-0.0813
	(-4.7331)	(-1.1890)	(-3.7409)	(-1.2215)
H*old	0.1530***	0.00175*	0.0975***	0.0128*
	(11.0305)	(1.8852)	(9.8058)	(1.8995)
**constant**	16.5263***	8.9160***	14.4356***	8.7941***
	(8.8555)	(9.1114)	(7.8597)	(9.1071)
**Controls**	Yes	Yes	Yes	Yes
**Year FE**	Yes	Yes	Yes	Yes
**Individual FE**	Yes	Yes	Yes	Yes
**N**	651	651	651	651
**R** ^**2**^	0.9386	0.9213	0.9345	0.9213

Note: t-statistics in parentheses

***, **, and * indicate significant at 1%, 5%, and 10% significance levels. H denotes the quantity of education(edu) and quality of education(eduq), respectively.

Column (2) in Table 2 displays the results of the mitigating effect of education quality on the negative impact of ageing. There is a significant positive cross-multiplier term between ageing and the teacher-student ratio, suggesting that education quality protects labor productivity from the negative effects of ageing. Elevated education quality promotes economic growth, raises labor force earnings, and reduces the burden of old age. Boosting the returns to average years of schooling are higher as the quality of education improves. And in areas with high education quality, the labor force is more receptive to new technologies, which is conducive to economic growth. The quality of education can play a role in mitigating the negative impacts of ageing by raising individual returns, increasing incomes, and reducing the burden of old age.

Columns (3) and (4) in Table 2 use the replacement variable method to test the robustness of the results in columns (1) and (2). The moderating effects of quantity and quality of education are examined using the ratio of the population aged 65 and over to the working population aged 15–64 as a proxy indicator. The results show that the interaction terms with the quantity and quality of education, respectively, remain significantly positive after replacing the ageing indicator. The results in columns (1) and (2) are robust to the fact that the quantity and quality of education can mitigate the negative impact of ageing on labor productivity.

### 4.2 Endogeneity test

#### 4.2.1 Instrumental variable approach

Ageing not only affects labor productivity, but also is affected by the level of economic development. There is a reverse causality between ageing and labor productivity, and the regression results in Table 2 may lead to measurement bias due to the existence of endogeneity. The lagged term of birth is therefore utilized as an instrumental variable to solve the endogeneity problem under the condition of taking into account the endogeneity of ageing [35]. The birth rate with a lag of 30 years is chosen as the instrumental variable of ageing. The reasons are as follows. First of all, in terms of demography, 30 years is close to the generational replacement time, which can better reflect the impact of birth rate on the change of population age structure. Sixty years is too long, during which time there may be other difficult to control policies and other factors affecting the age structure of the population. Secondly, the correlation of tool variable selection is satisfied. The birth rate at the end of the 30-year lag period will directly affect the degree of ageing in the sample range. Then, the externality of instrumental variable selection is satisfied. Labor productivity does not affect birth rates 30 years ago, reducing the possibility of reverse causality. Finally, after 30 years, systematic bias such as the measurement error of the recent time of the sample is also excluded. The regression results of the 2SLS model are displayed in Table 3. The regression results of the amount and quality of schooling, taking endogeneity into account, are shown in columns (1) and (2) in Table 3, respectively. The results of the first stage regression show that the birth rate in the lag period of 30 years is negatively correlated with ageing. The higher the birth rate, the less ageing degree, satisfying the condition of correlation between instrumental variables and endogenous variables. The Kleibergen-Pap rk Wald F-statistic indicates that it passes the test of weak instrumental variable. The results in Table 3 show that the cross-multiplier terms of ageing with education quantity and quality, respectively, continue to be significantly positive, subject to endogeneity considerations. This suggests that education quantity and quality can still reduce the negative effects of ageing.

**Table 3:** Regression considering endogeneity.

	(1)	(2)
	edu	eduq
**old**	-0.4726***	-0.3743***
	(-3.7928)	(-2.8758)
**H**	0.3714**	0.1485**
	(2.2693)	(2.24575)
old*H	0.2464***	0.0373**
	(7.6836)	(1.9820)
**First Stage**		
**birth (lag)**	-0.0849***	-0.0687***
	(-5.3938)	(-4.7965)
**First Stage F-stat**	19.97	19.93
**Controls**	Yes	Yes
**Year FE**	Yes	Yes
**Individual FE**	Yes	Yes
**Number of Observations**	651	651
**R** ^**2**^	0.9671	0.9566
**Kleibergen-Paap rk Wald F-stat**	27.126	24.485

Note: t-statistics in parentheses

***, **, and * indicate significant at 1%, 5%, and 10% significance levels. H denotes the quantity of education(edu) and quality of education(eduq), respectively.

#### 4.2.2 Space effects

Regression analysis of the spatial Durbin model according to Eq (2) begins with a spatial correlation test for each variable. The results show that the Moran index is not 0, indicating the existence of spatial correlation of the variables among regions. The results of the LR test show that the spatial Durbin model has better results than the spatial autoregressive model and the spatial error model. The existence of spatial correlation is based on the fact that either the inverse distance spatial weight matrix, the economic geospatial weight matrix or the economic spatial weight matrix that *σ*^2^ all pass the test, indicating that the model has a better fit. The findings of the spatial Durbin model regression are displayed in Table 4. Columns (1), (2) and (3) in Table 4 are regression results of education quantity, Columns (4), (5) and (6) are regression results of education quality, with columns (1) and (4) denoting the results of the model’s regression using the inverse distance spatial weight matrix, columns (2) and (5) denoting the model’s regression using the economic geospatial weight matrix, and columns (3) and (6) denoting the model’s regression using the economic spatial weight matrix.

**Table 4:** Spatial effects model.

	(1)	(2)	(3)	(4)	(5)	(6)
	edu			eduq		
**old**	-1.1279***	-1.0632***	-1.7087***	-0.4039***	-0.5291***	-0.1805***
	(-10.1588)	(-9.5758)	(-16.0782)	(-5.4529)	(-5.6833)	(-3.0466)
**H**	-0.7641***	-0.7045***	-1.2183***	-0.5072***	-0.8464***	-0.1715***
	(-7.9237)	(-7.2101)	(-12.5493)	(-5.0848)	(-7.0501)	(-2.9930)
old*H	0.1094***	0.1044***	0.1611***	0.0541***	0.0827***	0.0278***
	(10.5215)	(9.9718)	(16.5160)	(5.2409)	(6.3653)	(3.3571)
**Wx:**						
**old**	15.1341	-12.0030	0.1606	0.0009	0.5146**	-0.5581***
	(0.9728)	(-0.8611)	(0.5024)	(0.0046)	(2.2985)	(-3.2501)
**H**	-9.4760	-33.5367**	-0.0113	-0.2303	0.4932	-0.7911***
	(-0.6224)	(-2.3510)	(-0.0394)	(-1.0512)	(1.3863)	(-2.8644)
old*H	-1.3555	1.3481	0.0163	0.0179	-0.0784**	0.0828***
	(-0.9128)	(1.0090)	(0.4976)	(0.6405)	(-2.3204)	(3.3167)
**Spatial:**						
***ρ***	-9.9373***	-5.8683*	0.1663**	0.5448***	0.1937***	-0.1417*
	(-2.6853)	(-1.6766)	(2.0225)	(9.6302)	(2.8643)	(-1.8607)
**Variance:**						
***σ*** ^**2**^	0.2628***	0.2719***	0.2570***	0.1836***	0.2508***	0.2741***
	(18.0025)	(18.0213)	(17.9991)	(17.6721)	(18.0113)	(18.0202)
**N**	651	651	651	651	651	651
**R** ^**2**^	0.4467	0.6382	0.7786	0.6352	0.4619	0.5138

Note: t-statistics in parentheses

***, **, and * indicate significant at 1%, 5%, and 10% significance levels. H denotes the quantity of education(edu) and quality of education(eduq), respectively.

The results in Table 4 show that the cross-multiplier term between average years of schooling and ageing is significantly positive under the three spatial weight matrices, indicating that the quantity of education can still mitigate the negative impact of ageing when spatial effects are taken into account. Because of the bias in analyzing the impact of spatial effects directly, the structure of the spatial Durbin model calculated by the economic spatial weight matrix is decomposed into direct effects, indirect effects and total effects (see Table 5). The decomposition results show that the direct and total effects are significantly positive, indicating that the quantity of education can mitigate the negative impact of ageing in the region, and the indirect effect is positive but not significant. Similar spatial Durbin modeling results are obtained using the teacher-student ratio to represent human capital. After considering spatial factors, the quantity and quality of education can still mitigate the negative effects of ageing.

**Table 5:** Decomposition of spatial effects.

	Direct effect	Indirect effect	Total effect
old*edu	0.1618***	0.0505	0.2123***
	(16.1117)	(1.2510)	(5.1372)
old*eduq	0.0265***	0.0746***	0.1011***
	(3.1487)	(3.2934)	(4.5328)

Note:

***, **, * indicate significant at 1%, 5%, 10% level of significance.

### 4.3 Threshold panel model

Using a threshold panel model to test the impact of ageing on labor productivity under different levels of education quantity and education quality, the first step is to test the core variable is not time invariant [36]. The average values of ageing, average years of education and teacher-student ratio from 2000 to 2010 were 8.50, 8.02 and 6.62, respectively, and those from 2010 to 2020 were 10.39, 8.85 and 6.02, respectively. The mean varies significantly over time, so the core variable is not time invariant. The second step is to test for the existence of a threshold effect. Table 6 shows the results of the single threshold effect test. The results demonstrate that the P value of the test passes when the threshold variables are the average years of education and the teacher-student ratio, suggesting the existence of a threshold effect for both the quantity and quality of education. Average years of education is the threshold variable, the threshold value is 10.8681, and its 95% confidence interval is [10.6858, 10.9696]. Teacher-student ratio is a threshold variable with a threshold value of 7.7952 and a 95% confidence interval of [7.7524, 7.78601].

**Table 6:** Threshold effect tests.

threshold variable	F-value	P-value	BS frequency	Critical value		
				1%	5%	10%
**edu**	123.36	0.00	500	57.20	45.83	38.77
**eduq**	72.74	0.022	500	77.84	59.46	48.09

Columns (1) and (2) in Table 7 show the regression results of the single threshold panel model with average years of schooling and student-teacher ratio as threshold variables, respectively. Column (1) in Table 7 shows that ageing is detrimental to labor productivity when the average years of schooling is lower than 10.8681 years, and when the average years of schooling is higher than 10.8681 years, ageing starts to have a boosting effect on labor productivity. At present, the mean value of average years of schooling in China is 9.14 years, and the median is 9.11 years, which is about 2 years away from the threshold. The average years of education of the labor force in most regions is below 10.8681 years. There is a negative impact of ageing on labor productivity under the current conditions of average years of schooling. Column (2) in Table 7 shows that when the teacher-student ratio is lower than 7.7952, ageing can promote labor productivity, but when the teacher-student ratio is higher than 7.7952, ageing will have a negative impact on labor productivity. The quality of education mitigates the negative effects of ageing to the greatest extent possible when the student-teacher ratio is higher than 7.7952, i.e., when there are on average close to eight teachers for every 100 students. As the fertility rate decreases, the number of students attending school decreases and exceeding the optimal student-teacher ratio results in a waste of resources. Currently, China’s teacher-student ratio has a mean of 5.9 and a median of 5.74, with the teacher-student ratios in most regions well below the optimal level. The results in Table 5 show that when the average years of education exceeds 10.8681 and the teacher-student ratio reaches 7.7952, the quantity and quality of education can mitigate the negative impact of ageing on labor productivity.

**Table 7:** Threshold panel model regression results.

	(1)	(2)
	edu	eduq
**old(H≤γ)**	-0.0003	0.0451*
	(-0.0134)	(1.7179)
**old(H>γ)**	0.2116***	-0.1011***
	(6.4118)	(-3.5415)
**constant**	-2.4758**	3.2488***
	(-2.4055)	(3.8026)
**Controls**	Yes	Yes
**N**	651	651
**R** ^**2**^	0.9096	0.9009

Note: t-statistics in parentheses

***, **, and * indicate significant at 1%, 5%, and 10% significance levels. H denotes the quantity of education(edu) and quality of education(eduq), respectively.

### 4.4 Heterogeneity test

The role of the quantity and quality of education in mitigating the negative impacts of ageing may vary between regions due to significant differences in the level of economic development, the degree of ageing and the level of human capital between regions. Based on the level of economic development and geographic location, the 31 regions are divided into eastern, central, and western regions. The 31 regions are divided into southern and northern regions based on the Qinling-Huaihe River line, and the provinces crossed by the line are categorized as southern or northern regions according to their geographical area, population and economic center of gravity.

Table 8 presents the results of categorizing the 31 regions into three parts: east, central and west. Columns (1), (2) and (3) in Table 8 test the role of quantity of education in mitigating the negative effects of ageing in the eastern, central and western regions. The cross-multiplier term between average years of schooling and agieng is significantly positive in the eastern and western regions and insignificant in the central region. The degree of education has a mitigating effect on the negative consequences of ageing in the eastern and western regions, but it has no effect in the center region. The level of ageing in the eastern region is higher than that in the central and western regions, but the level of economic development and average years of education are higher than that in the central and western regions. In the service-oriented eastern region, higher average years of schooling can make up for the shortage of labor force, increase the effective labor supply, and facilitate economic development, thus alleviating the negative impact of ageing. In the western region, where the ageing level is lower, higher average years of schooling can improve the quality of the labor force and promote economic growth. Manufacturing predominates in the central region, but it is more negatively impacted by the labor supply drop and the average number of years of education increases more slowly and insufficiently to offset the negative effects of ageing. Columns (4), (5) (6) in Table 8, list the role of education quality in mitigating the negative impact of ageing in the eastern, central and western regions. The cross-multiplier terms of teacher-student ratio and ageing are significantly positive in the eastern and western regions and significantly negative in the central region. The eastern region has a higher level of economic development and a high demand for a highly qualified and innovative labor force, and therefore pays more attention to the quality of education. The policy favors the western area, allocates more resources to the west, and places a premium on education quality. Conversely, the central area places less value on high-quality education due to its dense population, high rate of ageing, and other factors.

**Table 8:** Tests of heterogeneity I.

	(1)	(2)	(3)	(4)	(5)	(6)
	edu			eduq		
	east	centra	west	east	centra	west
**old**	-1.5273***	0.1611	-1.3711***	-0.9309***	0.0311	-1.1640***
	(-4.8191)	(0.6690)	(-8.0853)	(-4.0133)	(0.9391)	(-8.6640)
**H**	-0.9992*	0.1031	-0.8821***	-1.2239***	0.2073	-1.6232***
	(-1.9493)	(0.3306)	(-4.6137)	(-3.3333)	(1.3906)	(-10.1179)
old*H	0.1421***	-0.0145	0.1863***	0.1197***	-0.0080	0.2171***
	(4.4694)	(-0.5348)	(10.4495)	(3.5356)	(-0.6276)	(11.5128)
**Controls**	Yes	Yes	Yes	Yes	Yes	Yes
**Year FE**	Yes	Yes	Yes	Yes	Yes	Yes
**Individual FE**	Yes	Yes	Yes	Yes	Yes	Yes
**N**	231	168	252	231	168	252
**R** ^**2**^	0.9452	0.9836	0.9578	0.9385	0.9854	0.9593

Note: t-statistics in parentheses

***, **, and * indicate significant at 1%, 5%, and 10% significance levels. H denotes the quantity of education(edu) and quality of education(eduq), respectively.

Table 9 presents the results of dividing the 31 districts into two parts, the southern and northern regions. Columns (1) and (2) in Table 9 test the role of quantity of education in mitigating the negative effects of ageing in both the northern and southern regions. Mean years of schooling and ageing in cross-country are significantly positive in both the southern and northern regions. A high level of education mitigates the negative effects of ageing in both the southern and northern regions. Columns (3) and (4) in Table 9 describe the role of quality of education in mitigating the negative effects of ageing in both northern and southern regions. The cross-multiplier term of student-teacher ratio and ageing is insignificant in the northern region and significantly positive in the southern region. The quality of education mitigates the negative effects of ageing in the south but fails to do so in the north.

**Table 9:** Heterogeneity test II.

	(1)	(2)	(3)	(4)
	edu		eduq	
	north	sourth	north	sourth
**old**	-1.5313***	-0.2327	-0.2031	-0.4209***
	(-8.8434)	(-1.6293)	(-1.2905)	(-4.3467)
**H**	-0.5689**	-0.4643**	-0.3850**	-1.0341***
	(-2.5462)	(-2.5706)	(-2.2112)	(-7.0459)
old*H	0.1634***	0.0375**	0.0316	0.0907***
	(9.3721)	(2.5659)	(1.6170)	(6.0370)
**Controls**	Yes	Yes	Yes	Yes
**Year FE**	Yes	Yes	Yes	Yes
**Individual FE**	Yes	Yes	Yes	Yes
**N**	315	336	315	336
**R** ^**2**^	0.9521	0.9756	0.9391	0.9787

Note: t-statistics in parentheses

***, **, and * indicate significant at 1%, 5%, and 10% significance levels. H denotes the quantity of education(edu) and quality of education(eduq), respectively.

## 5. Conclusion

China has become an ageing country with a low fertility rate and a longer life expectancy. Future economic development will take place under conditions of ageing with fewer children. Ageing before wealth makes the impact of ageing on economic growth more severe in China than in other countries. Under the condition of ageing, it is important to increase labor productivity in order to achieve common wealth and promote economic development. Human capital is an important factor affecting labor productivity. We focus on whether the quantity and quality of education can mitigate the negative impact of ageing on labor productivity, and introduces the cross-multiplier terms of the quantity and quality of education and ageing respectively for verification. The results show that the quantity and quality of education can significantly mitigate the negative impact of ageing. And when the average years of education exceeds 10.8681 and the teacher-student ratio reaches 7.7952, the quantity and quality of education can mitigate the negative impact of ageing more significantly. The results of the heterogeneity test show that the quantity and quality of education can mitigate the negative effects of ageing in both the eastern and western regions. The quality of education mitigates the negative effects of ageing only in the southern region and has not yet played a role in mitigating the negative effects of ageing in the northern region.

Based on the analysis of the relationship between ageing, quality of education and quantity of education, and labor productivity, the following insights have been gained. First, the number of years of free education should be extended to further raise the average number of years of education. The average number of years of education in China’s current labor force is 9.14 years, and although the quantity of education can alleviate the negative impact of ageing, there is still a gap of nearly two years from the threshold of 10.8681 years to compensate for the negative impact of ageing. Extending the number of years of free education promotes the average number of years of education above the threshold. Second, increase public investment in education, increase the teacher-student ratio, and promote the quality of education. The quality of education can mitigate the negative effects of ageing. When the teacher-student ratio reaches 7.7952, that is, eight teachers per 100 students, it can better mitigate the negative effects of ageing. China currently has less than 6 teachers per 100 students, and further increasing public investment in education can maximize the role of human capital. Third, the imbalance in economic development between regions has improved the quality of education in the north. The northern region is dominated by manufacturing and heavy industry, and development is based on traditional factor inputs that are both inefficient and polluting to the environment. Northern regions should emphasize public education inputs to improve technological progress and promote industrial transformation and upgrading.

## Supporting information

S1 Data (XLSX)
